# How should we appropriately classify low-risk uterine cervical cancer patients suitable for de-intensified treatment?

**DOI:** 10.1093/jrr/rrab130

**Published:** 2022-01-18

**Authors:** Naoya Murakami, Ikumi Kuno, Hiroshi Yoshida, Kouya Shiraishi, Tomoyasu Kato, Hiroshi Igaki

**Affiliations:** Department of Radiation Oncology, National Cancer Center Hospital, Tokyo, 104-0045, Japan; Department of Gynecologic Oncology, National Cancer Center Hospital, Tokyo, 104-0045, Japan; Division of Genome Biology, National Cancer Center Research Institute, Tokyo, 104-0045, Japan; Department of Diagnostic Pathology, National Cancer Center Hospital, Tokyo, 104-0045, Japan; Division of Genome Biology, National Cancer Center Research Institute, Tokyo, 104-0045, Japan; Department of Gynecologic Oncology, National Cancer Center Hospital, Tokyo, 104-0045, Japan; Department of Radiation Oncology, National Cancer Center Hospital, Tokyo, 104-0045, Japan

**Keywords:** low-risk, human papillomavirus (HPV), de-intensification, de-escalation, uterine cervical cancer

## Abstract

We suggested de-escalation would be possible for cervical cancer like human papillomavirus (HPV)-related oropharyngeal cancer. However, the classification was based on tumor shrinkage that can be obtained after half of the treatment was finished. Our other article found adverse factors which can be obtained prior to treatment, and they might classify patients earlier.

It is reported that about 70% of oropharyngeal cancers are related to human papillomavirus (HPV) infection and are radiosensitive. Therefore, recently de-escalation of treatment intensity has been attempted [1, 2].

It is known that the proportion of HPV-related tumors is much higher in uterine cervical cancer [3]. However, such de-intensification has not been attempted in cervical cancer management. There is a review article against the idea of de-escalation for cervical cancer [4]. Meanwhile, our group recently published an article in which low-risk patients can be controlled by lower doses, suggesting the possibility of de-intensification in cervical cancer patients [5]. In this article, the definition of low-risk was defined as follows: (i) squamous cell carcinoma, (ii) tumor shrinkage calculated by initial and before the first brachytherapy tumor size <26%, (iii) tumor size before the first brachytherapy <4 cm, and (iv) total treatment time < 9 weeks. However, the tumor shrinkage and size before the first brachytherapy are obtained only after half of the treatment is finished. If risk classification can be performed prior to treatment, it would be more helpful since brachytherapy sessions can be scheduled beforehand, especially for high-volume centers in which it is necessary to schedule multi-fractions for many patients.

Recently, our group published another article in which *TP53* mutations and non-HPV16/18 genotypes were adverse prognostic clinical factors [6]. Additionally, Miyasaka *et al.* reported that cervical adenocarcinoma patients who had CD8-positive tumor-infiltrating lymphocytes in the tumor nests had significantly better overall survival [7]. The authors now hypothesize that using such pathological factors that can be obtained before treatment, we can identify low-risk patients much earlier and can efficiently make treatment schedules prior to treatment.

Recently, results of IntErnational study on MRI-guided BRAchytherpay in CErvical cancer (EMBRACE-I) involving over 1300 patients has been reported that can be used as a benchmark for clinical practice in image-guided adaptive brachytherapy (IGABT) for cervical cancer [8]. In the protocol, it was recommended to deliver high-risk CTV (CTV_HR_) D_90_ ≥ 85 Gy EQD_2_. Although 25% of the patients received CTV_HR_ D_90_ < 85 Gy EQD_2_, 183 patients (14.6%) developed grade 3–5 late toxicities, which is somewhat higher than the level of rate of late toxicities in Japanese daily practice [9–11]. It is often the case that clinical outcomes of IGABT from Japan are underrecognized since the prescription goal of the Japanese guidelines is lower than 85 Gy EQD_2_ due to adopting central shielding (CS) after 30 Gy–40 Gy of pelvic external beam irradiation to protect the rectum and bladder from higher radiation exposure. However, obtained clinical outcomes themselves are not so worse or comparable than that of Europa or the United States. Although the central 3 cm–4 cm of the pelvis does not receive direct radiation, CS delivers a non-negligible dose to the tumor [12], and total treatment time is reduced because brachytherapy begins during external beam radiation therapy when CS is used. These points may play a supportive role in the positive clinical outcomes of the Japanese clinical series. Furthermore, it is supposed that the existence of the low-risk group is the reason why a lower dose works in the management of cervical cancer. Despite lower median CTV_HR_ D_90_ of 69 Gy (range 63 Gy–74 Gy), Kusada *et al.* recently reported favorable 2-year local control of > 90% for patients with CTV_HR_ D_90_ ≥ 70 Gy EQD_2_ or overall treatment time < 57 days in definitive radiotherapy involving IGABT and whole pelvic radiotherapy without CS [13]. This finding also confirms the existence of low-risk cervical cancer patients.

While it is true that some patients require a higher dose as to >85 Gy EQD_2_, the authors think that now it is high time for uterine cervical cancer management to change from a one-size-fits-all strategy to a more personalized and sophisticated treatment strategy.

## CONFLICT OF INTEREST

Dr. Igaki reports grants and personal fees from HekaBio, grants from CICS, grants from Elekta KK, personal fees from AstraZeneca, personal fees from Itochu, personal fees from HIMEDIC, personal fees from Varian, outside the submitted work. The others receive no financial support from any third party.
